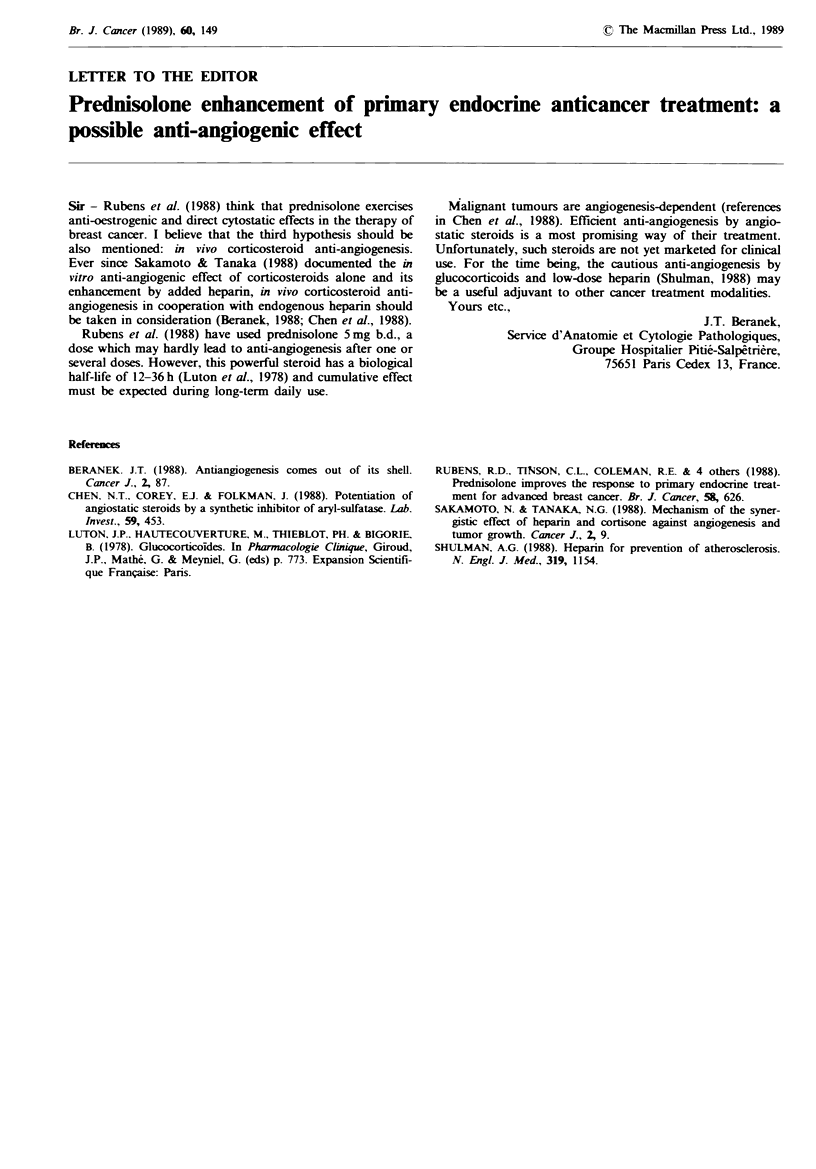# Prednisolone enhancement of primary endocrine anticancer treatment: a possible anti-angiogenic effect.

**DOI:** 10.1038/bjc.1989.238

**Published:** 1989-07

**Authors:** J. T. Beranek


Br. J. Cancer (1989), 60, 149 © The Macmillan Press Ltd., 1989

LETTER TO THE EDITOR

### Prednisolone enhancement of primary endocrine anticancer treatment: a possible anti-angiogenic effect

Sir – Rubens *et al.* (1988) think that prednisolone exercises anti-oestrogenic and direct cytostatic effects in the therapy of breast cancer. I believe that the third hypothesis should be also mentioned: *in vivo* corticosteroid anti-angiogenesis. Ever since Sakamoto & Tanaka (1988) documented the *in vitro* anti-angiogenic effect of corticosteroids alone and its enhancement by added heparin, *in vivo* corticosteroid anti-angiogenesis in cooperation with endogenous heparin should be taken in consideration (Beranek, 1988; Chen *et al.*, 1988).

Rubens *et al.* (1988) have used prednisolone 5 mg b.d., a dose which may hardly lead to anti-angiogenesis after one or several doses. However, this powerful steroid has a biological half-life of 12–36 h (Luton *et al.*, 1978) and cumulative effect must be expected during long-term daily use.

Malignant tumours are angiogenesis-dependent (references in Chen *et al.*, 1988). Efficient anti-angiogenesis by angiostatic steroids is a most promising way of their treatment. Unfortunately, such steroids are not yet marketed for clinical use. For the time being, the cautious anti-angiogenesis by glucocorticoids and low-dose heparin (Shulman, 1988) may be a useful adjuvant to other cancer treatment modalities.

Yours etc.,

J.T. Beranek,
Service d'Anatomie et Cytologie Pathologiques,
Groupe Hospitalier Pitié-Salpêtrière,
75651 Paris Cedex 13, France.

**References**

BERANEK, J.T. (1988). Antiangiogenesis comes out of its shell. *Cancer J.*, **2**, 87.

CHEN, N.T., COREY, E.J. & FOLKMAN, J. (1988). Potentiation of angiostatic steroids by a synthetic inhibitor of aryl-sulfatase. *Lab. Invest.*, **59**, 453.

LUTON, J.P., HAUTECOUVERTURE, M., THIEBLOT, PH. & BIGORIE, B. (1978). Glucocorticoïdes. In *Pharmacologie Clinique*, Giroud, J.P., Mathé, G. & Meyniel, G. (eds) p. 773. Expansion Scientifique Française: Paris.

RUBENS, R.D., TINSON, C.L., COLEMAN, R.E. & 4 others (1988). Prednisolone improves the response to primary endocrine treatment for advanced breast cancer. *Br. J. Cancer*, **58**, 626.

SAKAMOTO, N. & TANAKA, N.G. (1988). Mechanism of the synergistic effect of heparin and cortisone against angiogenesis and tumor growth. *Cancer J.*, **2**, 9.

SHULMAN, A.G. (1988). Heparin for prevention of atherosclerosis. *N. Engl. J. Med.*, **319**, 1154.